# Development and Validation of a Hypoglycemia Risk Model for Intensive Insulin Therapy in Patients with Type 2 Diabetes

**DOI:** 10.1155/2020/7292108

**Published:** 2020-09-19

**Authors:** Xiling Hu, Weiran Xu, Shuo Lin, Cang Zhang, Cong Ling, Miaoxia Chen

**Affiliations:** ^1^Department of Medicine, The Third Affiliated Hospital of Sun Yat-sen University, Guangzhou 510630, China; ^2^School of Nursing, Sun Yat-sen University, Guangzhou 510085, China; ^3^Department of Endocrinology and Metabolism, The Third Affiliated Hospital of Sun Yat-sen University, Guangzhou 510630, China; ^4^Department of Neurosurgery, The Third Affiliated Hospital of Sun Yat-sen University, Guangzhou 510630, China; ^5^Nursing Department, The Third Affiliated Hospital of Sun Yat-sen University, Guangzhou 510630, China

## Abstract

**Aims:**

To develop a simple hypoglycemic prediction model to evaluate the risk of hypoglycemia during hospitalization in patients with type 2 diabetes treated with intensive insulin therapy.

**Methods:**

We performed a cross-sectional chart review study utilizing the electronic database of the Third Affiliated Hospital of Sun Yat-sen University, and included 257 patients with type 2 diabetes undergoing intensive insulin therapy in the Department of Endocrinology and Metabolism. Logistic regression analysis was used to derive the clinical prediction rule with hypoglycemia (blood glucose ≤ 3.9 mmol/L) as the main result, and internal verification was performed.

**Results:**

In the derivation cohort, the incidence of hypoglycemia was 51%. The final model selected included three variables: fasting insulin, fasting blood glucose, and total treatment time. The area under the curve (AUC) of this model was 0.666 (95% CI: 0.594–0.738, *P* < 0.001).

**Conclusions:**

The model's hypoglycemia prediction and the actual occurrence are in good agreement. The variable data was easy to obtain and the evaluation method was simple, which could provide a reference for the prevention and treatment of hypoglycemia and screen patients with a high risk of hypoglycemia.

## 1. Introduction

The prevalence of diabetes has increased significantly in recent decades and was estimated to be about 116.4 million in 2019 in China [1]. However, only 25.8% patients received treatment for diabetes, and only 39.7% of those treated had adequate glycemic control [2].

Type 2 diabetes mellitus (T2DM) is characterized by a progressive deterioration of *β*-cell function and reduction in insulin sensitivity [3, 4]. Several studies have shown that intensive insulin therapy can improve the function of islet *β*-cells, alleviate clinical symptoms of some patients, reduce diabetes-related complications, and improve the long-term prognosis of patients with T2DM [5, 6]. Indications for intensive insulin therapy in hospitalized patients are newly diagnosed T2DM patients with obvious hyperglycemia symptoms and patients who have not reached the standard (HbA1c > 7%) after adequate drug dose adjustment [7].

Hypoglycemia is one of the most frequent adverse events in intensive insulin therapy for patients with T2DM [8]. Hypoglycemia leads to discomfort, reduced medication compliance and quality of life, and increased medical costs [9]. Severe hypoglycemia can cause damage to multiple organ functions resulting in cardiovascular and cerebrovascular diseases, loss of consciousness, and even death [10]. Patients with a high risk of developing hypoglycemia will benefit from lower intensive treatment and less stringent glucose level goals [11]. Therefore, assessing the risk of hypoglycemia is an important aspect of intensive insulin therapy.

However, few studies have reported predictive factors and risk factors for hypoglycemia in hospitalized patients with T2DM treated with intensive insulin therapy. An objective and simple clinical prediction rule may help guide more appropriate insulin dose setting and hypoglycemia prevention. Therefore, we aimed to establish a hypoglycemia risk model for hospitalized patients with T2DM treated with intensive insulin therapy in China, identify the risk factors of hypoglycemia as soon as possible, and take active measures to reduce the incidence of hypoglycemia and improve the physical and mental health of such patients.

## 2. Materials and Methods

### 2.1. Patient Identification and Eligibility

We performed a cross-sectional chart review study utilizing the electronic database of the Third Affiliated Hospital of Sun Yat-sen University. Adult patients with diabetes who were seen in the Department of Endocrinology and Metabolism between January 1, 2016, and December 30, 2018, were eligible if they were on intensive insulin treatment.

### 2.2. Baseline Predictor Variables and Outcome Measures

Based on the review of previous studies, we collected the following clinical data from hospitalized patients: sex, age, education, body mass index (BMI), waist-hip ratio, smoking history, course of disease, treatment before admission, glycated hemoglobin A1c (HbA1c), fasting blood glucose (FBG), random blood glucose (RBG), postprandial blood glucose (PBG), total insulin dose, total treatment time, combined oral antidiabetic drugs, fasting insulin, fasting C-peptide, diabetic nephropathy, diabetic retinopathy, diabetic peripheral nerve disease, diabetic peripheral vascular disease, hypertension, hyperlipidemia, cardiovascular disease, cerebrovascular disease, fatty liver, aspartate aminotransferase (AST), glutamic-pyruvic transaminase (ALT), blood urea nitrogen (BUN), creatinine (Cr), uric acid (UA), total cholesterol (TC), triglyceride (TG), high-density lipoprotein cholesterol (HDL-C), and low-density lipoprotein cholesterol (LDL-C). Patients with blood glucose levels ≤ 3.9 mmol/L during hospitalization were included in the hypoglycemic group; otherwise, they were included in the nonhypoglycemic group.

### 2.3. Statistical Analyses

We used SPSS 21.0 Statistics (IBM, USA) for data analysis. Normally distributed continuous data were described by mean and standard deviation. Independent *t*-test was used for intergroup comparison. The discrete data were described by frequency, composition ratio, or percentage, and the chi-square and Mann–Whitney *U* tests were used. A logistic regression model was constructed, with hypoglycemia as an independent result. Stepwise logistic regression was applied to the factors with a single-factor logistic regression *P* value < 0.10 to screen for hypoglycemia risk factors. Patient data were substituted into the logistic regression equation to calculate the predicted probability of hypoglycemia for each patient in the verification library. Receiver operating characteristic (ROC) curve was used to predict probability. And bootstrap method was used for internal verification. According to the predicted probability of hypoglycemia and the actual occurrence of hypoglycemia, the Hosmer–Lemeshow test was used to verify the calibration degree of the established logistic regression model.

## 3. Results

### 3.1. General Conditions in the Hypoglycemic Group and the Nonhypoglycemic Group

A total of 257 patients were included in this study, and 131 patients suffered from hypoglycemia (51.0%) during treatment. The results of FBG, total insulin dose, total treatment time, fasting C-peptide, fasting insulin, ALT, and Cr were significantly different between the two groups (*P* < 0.05) (Table 1).

### 3.2. Results of Logistic Regression Analysis

Univariate logistic regression factors with a *P* value < 0.10 were eligible for multivariable logistic regression. The factors that were ultimately entered into the model were fasting insulin, FBG, and total treatment time. The model was statistically significant (likelihood ratio chi − square = 19.892, degree of freedom = 3, and *P* < 0.001), and Akaike information criterion (AIC) was 270.89. Among them, the total treatment time ≥ 7 days was a risk factor for hypoglycemia, and FBG ≥ 7 mmol/L and fasting insulin ≥ 9.30 mu/L were protective factors for hypoglycemia (Table 2).

### 3.3. Internal Verification of Logistic Regression Model

**Figure 1:**
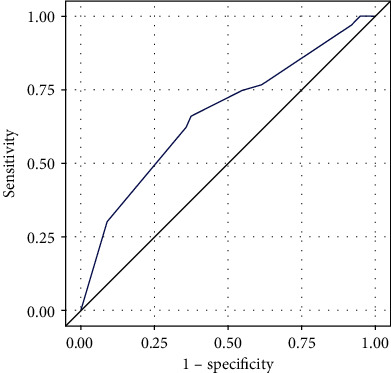
ROC curve of hypoglycemia probability in patients.

Substituting the patient data into the above logistic regression equation, the probability of hypoglycemia was 17–73%, and the risk of hypoglycemia was divided into “low” and “high” based on the probability of occurrence (Table 3). Using ROC curve to analyze the prediction probability (Figure 1), AUC = 0.666 (95% CI: 0.594–0.738; *P* < 0.001), the difference was statistically significant. In the 1,000 bootstrap datasets, the average of AUC was 0.664; there was no overfitting of the model. According to the predicted probability and the actual occurrence of hypoglycemia, the *P* value of the Hosmer–Lemeshow goodness-of-fit statistic with 5 degrees of freedom was 0.3662, and the chi-square was 5.4257. The prediction of hypoglycemia was in good agreement with the actual occurrence.

### 3.4. Rules for Predicting Hypoglycemia Risk Based on the Model

According to the model, the hypoglycemic risk prediction rules are formed (Table 4), Two or three evaluation results were “yes,” in which case the risk was rated as “high risk.” Two or three evaluation results were “no,” in which case the risk was rated as “low risk.”

## 4. Discussion

We developed a clinical prediction tool to identify the risk of hypoglycemia in patients with T2DM who undergo insulin intensive therapy. The accuracy of the prediction tool is fair. The area under the ROC is 0.666, which means that there is a 66.6% probability that the patients at risk of hypoglycemia will be correctly identified. With the addition of three independent variables including total treatment time ≥ 7 days, FBG < 7 mmol/L, and fasting insulin ≤ 9.30 mu/L, the risk of hypoglycemia gradually increased.

Our study found that the incidence of hypoglycemia in hospitalized patients with T2DM with intensive insulin therapy was 51.0%. The most common period of hypoglycemia was before lunch (23.6%), followed by after breakfast (21.5%) and before breakfast (19.9%). This was similar to the results of Pazos-Couselo et al., who found that the incidence of hypoglycemia was 50.8%, and the most common period was before lunch (32.0%) [12]. This was slightly higher than the 42.3% in the global HAT study [13]. In addition to the influence of insulin itself, prelunch hypoglycemia is related to the eating habits of the Chinese. First, the breakfast diet structure is generally based on staple food (such as steamed bread or porridge), which lacks protein and fat; hence, the digestion and absorption are relatively quick to happen. Second, it is also related to the fact that hospitalized patients need to undergo various tests in a fasted state. Simple meals after the tests cause hypoglycemia before lunch in some patients. The inability to correctly assess calorie intake has been identified as the root cause of hypoglycemia in hospitalized patients [14].

Furthermore, FBG ≥ 7 mmol/L and fasting insulin ≥ 9.30 mu/L are protective factors for hypoglycemia, which has not been reported in other studies and may be because of the specificity of intensive insulin therapy. The duration of hospitalization for intensive insulin therapy is relatively short, with an FBG of 4.4–7.0 mmol/L and random blood glucose (RBG) of ≤10 mmol/L as the control target [15]. Fasting blood glucose is a simple and cost-effective indicator for evaluating blood glucose levels, and patient blood glucose levels are very helpful in predicting hypoglycemia. Some studies pointed out that mean blood glucose, nadir blood glucose, and blood glucose coefficient of variation were predictors of hypoglycemia [16, 17]. Liu et al. showed that FBG was closely related to the recovery of first-phase insulin secretion in T2DM patients [18]. By observing the fluctuation characteristics of FBG, the probability of hypoglycemia can be predicted. At lower blood glucose levels, the sympathetic adrenal response is activated, and repeated hypoglycemic attacks can lead to impaired defense function, increasing the risk of severe hypoglycemia and forming a vicious cycle [19]. Patients with higher FBG levels are less likely to develop hypoglycemia.

FBG and fasting insulin reflect the level of insulin resistance. In the early stage of intensive insulin therapy, patients have obvious insulin resistance and islet cell dysfunction. Studies have shown that intensive insulin therapy can simulate the physiological secretion of human insulin, reduce the burden of pancreatic islet *β*-cells [20], and improve the T2DM insulin resistance and islet cell disorders [15]. Most patients who receive intensive insulin therapy are either newly diagnosed or have a more serious condition than other hospitalized patients, and the changes in islet function may hence be more obvious. Evaluating the changes in pancreatic islet function during treatment and timely adjustment of the drug dosage in patients who are sensitive to insulin therapy can reduce the occurrence of hypoglycemia. Malkani and Kotwal have a similar view, wherein they explained the higher incidence of hypoglycemia in young patients with T2DM as a result of higher insulin sensitivity [17].

Our study also found that the total treatment time of ≥7 days is an independent risk factor for hypoglycemia. Hypoglycemia interacts with the length of hospital stay, and hypoglycemia is related to the prolonged hospital stay [21, 22]. At the same time, the length of hospital stay is also used as an indicator of the risk of hypoglycemia in T2DM patients [23]. The interpretation is that the more serious the patient's condition, the longer the hospital stay. However, in our study, the insulin intensive treatment cycle is usually 14 days long. We believe that the increased risk of hypoglycemia may also be related to the gradual recovery of islet function in the later stage of treatment. Both explanations suggest that the medical team should reassess the patient's condition and adjust the insulin dose when the patient is treated for 7 days. The nurse should explain this to patients, reinforce hypoglycemia education, and manage carbohydrate intake [24].

Similar to our study, Ena et al. derived a predictive model including four indicators: glomerular filtration rate, insulin dose, length of hospital stay, and episodes of hypoglycemia during the previous 3 months [23]. Shah et al. developed a hypoglycemic predictive scoring tool for inpatients with diabetes and included five variables: age, emergency department visit in the previous 6 months, insulin use, use of oral agents that do not induce hypoglycemia, and severe chronic kidney disease [25]. Similarly, predictors of the risk of severe hypoglycemia in outpatients with diabetes included kidney disease, age, insulin use, sulfonylurea use, history of hypoglycemia, and history of emergency or hospitalization related to hypoglycemia [26, 27].

Chronic kidney disease is a well-known risk factor for hypoglycemia [28, 29], but the benefit of intensive insulin therapy for such patients is low. In our study, high serum Cr was only identified as a risk factor for hypoglycemia in the univariate analysis and was excluded from the multifactor model. We also excluded insulin dose, fasting C-peptide, and ALT. As the factors that cause hypoglycemia are interdependent in T2DM, it is very difficult to describe them separately [17].

Elderly patients suffer from decreased physiological functions. When hypoglycemia occurs, the body cannot effectively regulate blood glucose levels in a timely manner. At the same time, due to their generally prolonged disease course, they are often accompanied by neuropathy and hypoglycemia-associated autonomic failure. When the blood glucose level drops, the sympathetic nervous system cannot be effectively excited, and asymptomatic hypoglycemia is prone to occur [30]. There are also studies that use the course of diabetes as a predictor of hypoglycemia in hospitalized patients [31]. In addition, elderly patients have decreased kidney and liver metabolic function and poor drug clearance and are hence prone to hypoglycemia during intensive insulin therapy [32]. However, the study by Li et al. concluded that age ≥ 75 years reduced the risk of hypoglycemia, which was related to the relaxation of blood sugar control goals [33]. The age of the patients in our study was not statistically significant, which may be because of the relatively simple age stratification.

Regarding the effect of oral antidiabetic drugs on hypoglycemia, some studies showed that the use of sulfonylureas and biguanides affected the occurrence of hypoglycemia in patients with T2DM [26, 34, 35]. Due to the large amount of insulin administration in our patients, oral antidiabetic drugs had little effect on hypoglycemia, so they did not enter the model. Chow et al. used the ACCORD database to assess the risk of long-term severe hypoglycemia in T2DM and found that hypertension and the use of antihypertensive drugs were the influencing factors of hypoglycemia [35]. This may be related to the population of the study. Riddle included patients with high cardiovascular disease risk and abnormal blood sugar and reached similar conclusions [36]. In our study, hypertension and other cardiovascular diseases were not statistically significant.

The relatively small sample size and single-center setting are some of the limitations of our study. The influencing factors that cause hypoglycemia in hospitalized patients are complex. We wanted to comprehensively analyze the variables to deduce the prediction rules for hypoglycemia; however, owing to our limited research power, we could not evaluate the blood glucose status of patients before admission or compare different treatment options. In order to simplify the prediction rules, we did not include the patient's blood glucose variability [37] into the evaluation index. In this study, we did not assess and analyze the situation of severe hypoglycemia, because we believe that any degree of hypoglycemia should be taken seriously and intervened [38]. As our research has not been verified by external data, the results cannot be directly applied to the clinic. We plan to conduct verification studies in the future with larger patient samples and in a multicenter setting.

## 5. Conclusions

We proposed a model for predicting hypoglycemia risk in patients with T2DM treated with intensive insulin therapy. The variable data are easy to obtain and the evaluation method is simple, which can provide a reference for prevention and treatment of hypoglycemia and screen patients with high risk of hypoglycemia. The research results suggest that the actual clinical work should carefully establish blood glucose control goals; comprehensively evaluate the patient's FBG, fasting insulin, and total treatment time; adjust insulin dosage in time; and control blood glucose as efficiently as possible. Future research will focus on validating the risk model with external data in a larger cohort of patients with T2DM to help develop targeted interventions.

## Figures and Tables

**Table 1:** Comparison of the demographic and clinical characteristics of the hypoglycemic and nonhypoglycemic groups.

Patient characteristics	Hypoglycemic group	Nonhypoglycemic group	*t* or *Z* value	*P* value	Odds ratio, 95% confidence interval
Sex					
Male	86 (65.65)	86 (68.25)	0.197	0.657	1.12 (0.67, 1.89)
Female	45 (34.35)	40 (31.75)			
Age (years)					
≥60	61 (46.56)	53 (42.06)	0.527	0.468	1.20 (0.73, 1.96)
<60	70 (53.44)	73 (57.94)			
BMI (kg/m^2^)					
≥24	64 (48.85)	71 (56.35)	1.447	0.229	0.74 (0.45, 1.21)
<24	67 (51.15)	55 (43.65)			
Waist-hip ratio					
Central obesity	62 (47.69)	70 (55.56)	1.584	0.208	0.73 (0.45, 1.19)
Normal	68 (52.31)	56 (44.44)			
Course of disease (years)					
≥10	51 (38.93)	51 (40.48)	0.064	0.800	0.94 (0.57, 1.55)
<10	80 (61.07)	75 (59.52)			
HbA1c (%)					
≥7	117 (89.31)	115 (91.27)	0.280	0.597	0.80 (0.35, 1.83)
<7	14 (10.69)	11 (8.73)			
FBG (mmol/L)					
≥7	67 (51.15)	82 (65.08)	5.118	0.024^∗^	0.56 (0.34, 0.93)
<7	64 (48.85)	44 (34.92)			
Total insulin dose (IU)					
≥31	83 (63.36)	95 (75.40)	4.372	0.037^∗^	0.56 (0.33, 0.97)
<31	48 (36.64)	31 (24.60)			
Total treatment time (days)					
≥7	115 (87.79)	99 (78.57)	3.914	0.048^∗^	1.96 (1.00, 3.85)
<7	16 (12.21)	27 (21.43)			
Fasting C-peptide (nmol/L)					
>0.66	13 (10.48)	29 (23.77)	7.668	0.006^∗^	0.38 (0.18, 0.76)
≤0.66	111 (89.52)	93 (76.23)			
Fasting insulin (mu/L)					
>9.29	39 (29.77)	63 (50.00)	10.980	<0.001^∗^	0.42 (0.25, 0.71)
≤9.29	92 (70.23)	63 (50.00)			
Diabetic nephropathy					
Yes	29 (22.14)	29 (23.02)	0.028	0.866	1.05 (0.59, 1.89)
No	102 (77.86)	97 (76.98)			
Hypertension					
Yes	48 (36.64)	50 (39.68)	0.252	0.616	1.14 (0.69, 1.88)
No	83 (63.36)	76 (60.32)			
Cardiovascular disease					
Yes	42 (32.06)	44 (35.20)	0.283	0.595	1.15 (0.69, 1.93)
No	89 (67.94)	81 (64.80)			
AST (U/L)					
>40	9 (6.87)	12 (9.52)	0.603	0.438	0.70 (0.28, 1.73)
≤40	122 (93.13)	114 (90.48)			
ALT (U/L)					
>25	34 (26.15)	49 (40.50)	5.824	0.016^∗^	0.52 (0.31, 0.89)
≤25	96 (73.85)	72 (59.50)			
BUN (mmol/L)					
>8.2	12 (9.16)	16 (13.22)	1.051	0.305	0.66 (0.30, 1.46)
≤8.2	119 (90.84)	105 (86.78)			
Cr (*μ*mol/L)					
≥66	85 (64.89)	64 (51.20)	4.925	0.026^∗^	1.76 (1.07, 2.91)
<66	46 (35.11)	61 (48.80)			

^∗^ *P* < 0 : 05. BMI: body mass index; HbA1c: glycated hemoglobin A1c; FBG: fasting blood glucose; AST: aspartate aminotransferase; ALT: glutamic-pyruvic transaminase; BUN: blood urea nitrogen; Cr: creatinine.

**Table 2:** Multivariable logistic regression analysis of multiple factors.

	Regression coefficient (*β*)	Standard error of regression coefficient	Wald *χ*^2^	*P* value	Odds ratio, 95% confidence interval
Constant	-0.292	0.230	1.612	0.204	—
Fasting insulin ≥ 9.30 mu/L	-0.494	0.149	11.031	0.001	0.372 (0.208–0.667)
FBG ≥ 7.0 mmol/L	-0.341	0.153	4.972	0.026	0.505 (0.277–0.921)
Total treatment time ≥ 7 days	0.441	0.228	3.730	0.054	2.415 (0.987–5.908)

**Table 3:** Probability and risk level of hypoglycemia based on fasting insulin, FBG, and total treatment time.

Fasting insulin (mu/L)	FBG (mmol/L)	Total treatment time (days)	Probability of hypoglycemia (%)	Risk level
≥9.30	≥7.0	<7	17	Low
≥9.30	<7.0	<7	29	Low
≥9.30	≥7.0	≥7	33	Low
<9.30	≥7.0	<7	36	Low
≥9.30	<7.0	≥7	50	High
<9.30	<7.0	<7	53	High
<9.30	≥7.0	≥7	57	High
<9.30	<7.0	≥7	73	High

**Table 4:** Hypoglycemia risk assessment form for intensive insulin treatment of T2DM.

Items	Yes	No
Fasting insulin < 9.30 (mu/L)	□	□
FBG < 7 (mmol/L)	□	□
Total treatment time ≥ 7 days	□	□
Hypoglycemia risk	□high	□low

## Data Availability

The data used to support the findings of this study are available from the corresponding author upon request.
